# Gut Microbiome Modification through Dietary Intervention in Patients with Colorectal Cancer: Protocol for a Prospective, Interventional, Controlled, Randomized Clinical Trial in Patients with Scheduled Surgical Intervention for CRC

**DOI:** 10.3390/jcm11133613

**Published:** 2022-06-22

**Authors:** María Antonia Martínez-Sánchez, María Ángeles Núñez-Sánchez, Andrés Balaguer-Román, Alba Oliva-Bolarín, Gabriel Pujante-Gilabert, Quiteria Hernández-Agüera, María José Mesa-López, Juan Egea-Valenzuela, María Isabel Queipo-Ortuño, Antonio José Ruiz-Alcaraz, Mercedes Ferrer-Gómez, José Gil-Martínez, Bruno Ramos-Molina

**Affiliations:** 1 Obesity and Metabolism Laboratory, Biomedical Research Institute of Murcia (IMIB), 30120 Murcia, Spain; mariaantonia.martinez1@gmail.com (M.A.M.-S.); mangelesnunezsanchez@gmail.com (M.Á.N.-S.); a.balaguerroman@gmail.com (A.B.-R.); albaoliva99@gmail.com (A.O.-B.); tofly16@gmail.com (M.F.-G.); 2 Department of General and Digestive System Surgery, Virgen de la Arrixaca University Hospital, 30120 Murcia, Spain; gabrielpujante@yahoo.es (G.P.-G.); quihera@gmail.com (Q.H.-A.); 3 Unit of Gastrointestinal Endoscopy, Department of Digestive Diseases, Virgen de la Arrixaca University Hospital, 30120 Murcia, Spain; mari_ml_2@hotmail.com (M.J.M.-L.); juanegeavalenzuela@gmail.com (J.E.-V.); 4 Department of Medical Oncology, Virgen de la Victoria and Regional University Hospitals-IBIMA, UMA-CIMES, 29010 Malaga, Spain; maribelqo@gmail.com; 5 Department of Biochemistry, Molecular Biology B and Immunology, Faculty of Medicine, University of Murcia, 30100 Murcia, Spain; ajruiz@um.es; 6 Department of Endocrinology and Nutrition, Virgen de la Arrixaca University Hospital, 30120 Murcia, Spain

**Keywords:** colorectal cancer, gut microbiota, colorectal surgery, anastomotic leak, nutritional therapy

## Abstract

Colorectal cancer (CRC) is the third most common cancer and the second cause of cancer death worldwide. Several factors have been postulated to be involved in CRC pathophysiology, including heritable and environmental factors, which are the latest to be closely associated with nutritional habits, physical activity, obesity, and the gut microbiota. The latter may also play a key role in CRC prognosis and derived complications in patients undergoing surgery. This is a single-center, open, controlled, randomized clinical trial, in patients with scheduled surgical intervention for CRC. The primary objective is to assess whether a pre-surgical nutritional intervention, based on a high-fiber diet rich in polyunsaturated fatty acids (PUFAs), can reduce disturbances of the gut microbiota composition and, consequently, the rate of post-surgical complications in patients with CRC. Patients will be randomized in a 1:1 ratio after receiving a diagnosis of CRC. In the control arm, patients will receive standard nutritional recommendations, while patients in the intervention arm will be advised to follow a high-fiber diet rich in PUFAs before surgery. Participants will be followed up for one year to evaluate the overall rate of postsurgical complications, recurrences of CRC, response to adjuvant therapy, and overall/disease-free survival.

## 1. Introduction

The gut microbiota comprises a community of about 100 trillion microbes, including bacteria, viruses, and yeasts, that have a symbiotic and mutualistic relationship with the host [1]. Although the composition of the microbiota is believed to be fairly stable in adults, it is influenced not only by host genetics, but also by environmental factors including diet, chemical exposure, and antibiotic consumption. The role of the gut microbiota in health has been extensively explored, and together with microbial-derived metabolites, they play a critical role in several physiological processes including host metabolism, immune system, and even behavior [1,2,3]. Thus, the disruption of the normal balance of gut microbiota is characterized by the depletion of commensal bacteria, overgrowth of opportunistic pathogens, and reduction in total microbiota diversity [4]. Indeed, such disturbances can lead to the development of different disorders, ranging from metabolic disturbances to cardiovascular, neurological, respiratory, and intestinal conditions, and even certain types of cancer [5].

Colorectal cancer (CRC) represents the third most common cancer worldwide and it is the second leading cause of cancer death, with an associated high economic burden [6]. The exact etiology of CRC remains unclear, although several studies have focused on the evaluation of the mechanisms involved. Most CRC cases (approximately 90%) occur sporadically (not related to the genetics of family history of the disease), and several lifestyle factors including obesity, diets with a high content of fat and (or) red and (or) processed meat, environmental pollutants, cigarette smoking, and alcohol abuse, have been associated with CRC development and progression [7]. Furthermore, there is emerging evidence that patients with CRC display significant alterations in gut microbiota, mainly characterized by an increase in opportunistic pathogens (e.g., *Enterococcaceae*, *Campylobacter*) [8] and a decrease in butyrate-producing bacteria, including *Bifidobacteria*, *Roseburia* and *Faecalibacterium prausnitzii* [9]. Overall, these alterations in the gut microbiome have been proposed to play an important role in tumor formation and progression [7,10].

Currently, surgery represents the main treatment for patients with CRC [11]. Nonetheless, major postoperative complications are not unusual in these patients. These complications include anastomotic leakage, wound infection, ileus, and bleeding, which lead to an increase in the risk of morbimortality, length of hospital stay, and re-admission, which, in turn, increases the economic burden on health systems [12]. Remarkably, the gut microbiota and related metabolites have been described as key players in postoperative recovery. For instance, commensal bacteria are known to be involved in intestinal barrier healing and barrier function [13,14]. Thus, it is not surprising that disturbances of the gut microbiota in CRC patients undergoing surgery is related to postsurgical complications.

On the other hand, certain dietary factors have been suggested to be associated with CRC incidence, tumor progression, and response to therapy [15]. For instance, higher intakes of dietary fiber, dietary calcium, and yogurt have been associated with a lower risk of CRC [15]. Thus, nutritional strategies are emerging as a promising strategy for the prevention and management of CRC. In this direction, dietary fiber has been shown to modulate the gut microbiota profile, affecting metabolic activities in the gastrointestinal tract, favoring the growth of protective bacteria [16]. Importantly, diets rich in whole grains and dietary fiber have been associated with a lower risk of developing *F. nucleatum*-positive CRC [17]. In addition to dietary fiber, recent studies have highlighted the impact of dietary omega-3 polyunsaturated fatty acids (PUFAs) on the gut microbiota [18]. Moreover, PUFAs have been demonstrated to increase the proportion of butyrate bacterial genera, e.g., *Bifidobacterium*, *Roseburia* and *Lactobacillus* [19], while reducing the number of opportunistic pathogens leading to a reduction of microbial alterations in the gastrointestinal tract [20]. However, studies relating to the modulation of gut microbiota by diet and the outcomes related to CRC surgery are still scarce. Therefore, we outline here our study protocol for a study on the impact of a pre-surgical nutritional intervention based on the intake of a high-fiber diet rich in PUFAs on disturbances of the gut microbiota, and therefore, on post-surgical complications in patients with CRC undergoing elective surgery.

## 2. Methods

### 2.1. Study Objectives

The objective of this study is to evaluate whether a pre-surgical nutritional intervention based on a high-fiber diet rich in PUFAs can revert gut microbiota alterations, surgical site infections, and anastomotic leaks in patients with CRC undergoing elective surgery (colonic resection with anastomosis), compared to a control group following standard nutrition recommendations.

To achieve this objective, three major aims are defined:

1. To determine the change in the gut and intra-tumoral microbiome composition, fecal and serum levels of short-chain fatty acids (SCFAs), and serum inflammation, endotoxemia, and intestinal permeability markers in CRC patients following a high-fiber diet rich in PUFAs in the preoperative phase, in comparison to the control group.
2. To evaluate the rate of anastomotic dehiscence and surgical site infections after surgery (30 days of follow-up) in the intervention group, in comparison to the control group.
3. To establish the length of hospitalization, recurrences of CRC, response to adjuvant therapy, and overall and disease-free survival after surgery (1 year of follow-up) in the intervention group, in comparison to the control group.

### 2.2. Study Design

A single-center, parallel-arm, open, controlled, randomized clinical trial will be performed in patients with scheduled surgical intervention for CRC in the General Surgery and Digestive System Unit of the Virgen de la Arrixaca University Hospital (Murcia, Spain). All the included individuals must have passed the protocol of the Virgen de la Arrixaca University Hospital to be candidates for elective major surgery for CRC, and must sign the informed consent after being informed of the objectives and the methodology of the study.

### 2.3. Study Setting

Study subjects will be recruited in the General Surgery and Digestive System Unit of the Virgen de la Arrixaca University Hospital (Murcia, Spain) among those patients diagnosed with CRC and scheduled for elective surgery.

### 2.4. Eligibility Criteria

#### 2.4.1. Inclusion Criteria

Consecutive adult patients diagnosed with CRC and undergoing elective surgery will be eligible. The following criteria must be met for inclusion in the study:

1. Age between 18–80 years old;
2. After the clinical diagnosis of CRC (stage I–III), the doctor has recommended and arranged for CRC surgery.

#### 2.4.2. Exclusion Criteria

Patients who fulfill any of the following criteria will be excluded from the study:

1. Clinical diagnosis of stage IV CRC;
2. Current gastrointestinal illness other than gastroesophageal reflux disease or hemorrhoids;
3. Chronic liver or kidney disease;
4. History of cardiac disease;
5. Positive genetic test for inherited polyposis syndromes (such as familial adenomatous polyps, hereditary non-polyposis colon cancer syndromes, etc.);
6. Alcoholism or illicit drug use;
7. Antibiotic use within the past 2 months;
8. Dietary supplement use including pre- or probiotics within the past month;
9. History of intestinal cancer, inflammatory bowel disease, celiac disease, or malabsorptive bariatric surgery;
10. Inflammatory or connective tissue disease (such as lupus, scleroderma, rheumatoid arthritis, etc.).

### 2.5. Consent

Patient informed consent for participation will be obtained by the specialist or research nurse at the General Surgery and Digestive System Unit. A member of the team will inform patients of the objectives and methodology of the study and patients will be given a written Patient Information Sheet (Annex S1 in Supplementary Materials). Patients will be able to ask any questions regarding the Information Sheet. The Helsinki Declaration will be followed during the duration of the study.

### 2.6. Research Ethics Approval

This study will be carried out in the Virgen de la Arrixaca University Hospital in accordance with the current Spanish legislation that regulates the carrying out of biomedical research projects, for which this protocol is established as a reference document for review by the Ethical Committees, as well as for the practical decision-making in the management of included patients by participating researchers. The study has already obtained written authorization from the Research Ethics Committee (internal code 2021-2-7-HCUVA).

### 2.7. Protocol Amendments

Except for those emergency situations, no protocol changes or deviations will be allowed without documented approval. The Research Ethics Committee must be informed of possible changes and will approve in writing any change or deviation that may increase the risks of the subject and/or may adversely affect the rights of the volunteer or the validity of the research. This stipulation does not apply to those changes that are made to reduce the inconvenience or avoid risks to the subjects and to changes that would affect the administrative aspects of the study. This study will be conducted with respect for the rules of Good Clinical Practice (GCP) and the regulations and recommendations that appear in the Declaration of Helsinki and that are included in the current legislation on biomedical research at all times.

### 2.8. Intervention

**Figure 1:**
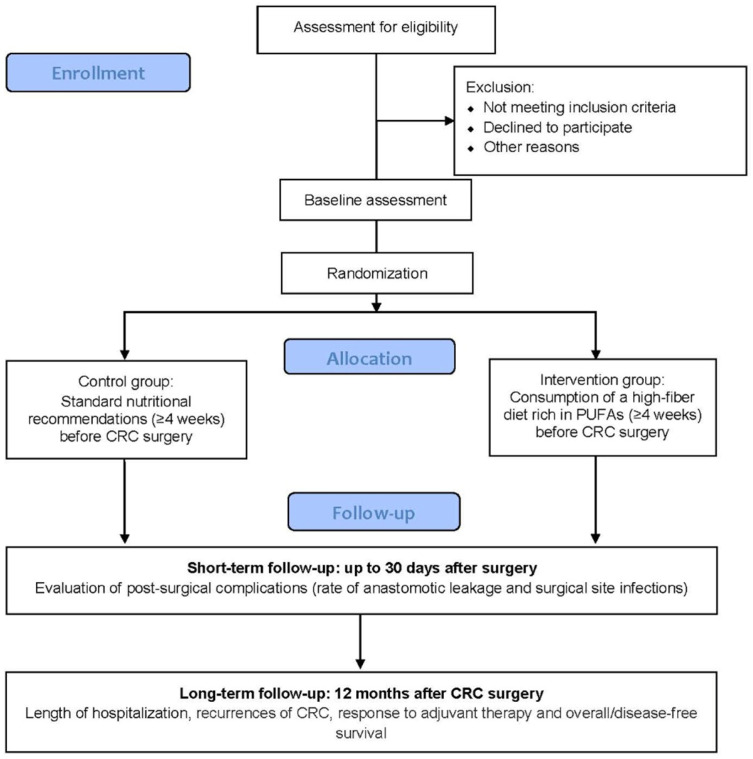
Trial flowchart of the intervention.

Patients will be randomized in a 1:1 ratio after receiving a diagnosis of CRC during endoscopic evaluation (Figure 1). Patients included in the control arm will receive standard nutritional recommendations, while patients in the intervention arm will be advised to follow a high-fiber diet rich in PUFAs (total dietary intake of at least 30 g of fiber per day, and at least 3 g of PUFAs per day from food, not supplements) for at least 4 weeks before surgery.

Participants may withdraw or revoke consent at any time without giving explanations and without any prejudice to them. Individuals who drop out of the study will not undergo further follow-up or be replaced by new subjects. Subjects may be withdrawn if researchers/clinicians consider that the participant can no longer comply with all the requirements of the study, or if any of the procedures is considered as potentially harmful to the subject.

As this is a prospective longitudinal study, some patients might find it difficult to follow the nutritional recommendations. Nevertheless, thanks to the close monitoring of the expert nutritionist, we will manage to reduce the abandonment percentage to a minimum. We plan to provide a written protocol in lay terms for the study participants, including a detailed handbook that they can use as a guide, and they will have full access to the nutritionist via telephone or weekly individual meetings.

Participants will not be allowed to consume any dietary supplement including pre- or probiotics during the intervention period.

### 2.9. Outcomes

The primary outcome includes the evaluation of changes in the gut and intra-tumoral microbiota composition and its relationship with the postoperative rate of anastomotic leakage and surgical site infection after the nutritional intervention. Secondary outcomes will include the determination of changes in SCFA levels in serum and feces, serum levels of inflammation markers, endotoxemia, and intestinal permeability, length of hospitalization after surgery, recurrence of CRC after surgery, response to adjuvant therapy, and post-surgical survival rates.

### 2.10. Participant Timeline

Individuals who agree to participate in this study will be assessed for eligibility. Eligible patients will be randomly assigned to the intervention or the control group (Figure 1). The trial will consist of a 4-week nutritional intervention pre-surgery, with a 12-month follow-up phase. The total trial period will be 13 months. A detailed assessment schedule can be found in Table 1.

### 2.11. Sample Size and Recruitment

Sample size calculation was based on expected differences in the rate of anastomotic dehiscence of CRC surgery. According to the literature, the anastomotic leak rate in CRC patients undergoing CRC surgery varies between 3% and 50%; therefore, we estimated that for a reduction in anastomotic leak from 30% to 10%, assuming a significance level of 5% and a β power of 0.8, 48 patients in each study group would be necessary. Considering a potential 10% of follow-up losses, 54 patients per group will be recruited.

### 2.12. Randomization and Blinding

Patients will be randomized in a 1:1 ratio after receiving a diagnosis of CRC in the endoscopic evaluation. In the control arm, patients will receive standard nutritional recommendations, while patients in the intervention arm will be advised to follow a high-fiber diet rich in PUFAs (total dietary intake of at least 30 g of fiber per day, and of at least 3 g of PUFAs per day from food, not supplements) at least 4 weeks before surgery. Simpler randomization will be computer-generated by a member of the study team at the site.

Given the nature of the intervention, neither patients nor research or medical staff will be blinded. Group assignment will be revealed to patients at the moment of participating acceptance.

### 2.13. Data and Sample Collection and Analysis of Variables

#### 2.13.1. Data Collection

Data and samples will be obtained at 3 different time points: at baseline, the day before CRC surgery, and one year after surgery. For data collection, a complete clinical evaluation of the patients will be performed, which will include the registration of anthropometric data (height, weight, body mass index (BMI), waist-to-hip ratio, blood pressure), information about the patient’s medication, and assessment of dietary intake using validated food frequency questionnaires (FFQs) [21]. Baseline dietary fiber and PUFA intake will be estimated from the FFQs. All participants will be asked to complete a food diary during the 4 weeks of intervention for assessment of dietary changes.

#### 2.13.2. Sample Collection and Storage

Tumor biopsies will be collected during both screening colonoscopy and elective CRC surgery, and will be immediately placed in a sterile collection tube. Samples will then be taken to the Anatomic Pathology Service of the Virgen de la Arrixaca University Hospital for fixation and standard evaluations.

For stool sample collection, participants will be asked to bring their samples at baseline, the day before the surgery, and at the follow-up visit. Patients will be given an appropriate sterile collection container. If the sample is collected the day before the appointment, the patient will be asked to place the sample in the freezer until the following day. Once the sample is collected by the research staff, it will be weighed and then placed in the fridge a maximum of 4 h prior to snap-freezing in N_2_, and stored at −80 °C.

For blood sample collection, patients will be asked to fast for 12 h before the appointment. A specialized nurse will obtain two samples of 7.5 mL, one in a collection tube with clot activator for serum and one in a collection tube with EDTA for plasma isolation. Plasma and serum samples will be used for the determination of endotoxemia, intestinal permeability and inflammation markers.

The analysis of SCFAs in plasma/feces will be performed by gas chromatography-triple quadrupole mass spectrometry (GC-QqQ/MS).

All samples will be stored frozen at −80 °C at the Biobank of IMIB (registered at the Spanish Biobank Register of the Institute of Health Carlos III, ref num. PT17/0015/0038).

#### 2.13.3. Analysis of Gut and Intra-Tumoral Microbiota Composition

Analysis of the gut and intra-tumoral microbiota composition will be performed by 16 s rRNA gene sequencing in stool and biopsy samples, respectively. DNA extraction from stool samples will be performed using 200 mg of sample using the QIAamp DNA Stool Mini kit (Qiagen, Manchester, UK), following the manufacturer’s instructions. DNA extraction from FFPE tissue samples will be performed on three sections of 10 μm using the QIAamp DNA FFPE Tissue Kit (Qiagen, Manchester, UK), following the manufacturer’s instructions. The concentration and quality of the DNA will be determined by the Agilent 2200 TapeStation system. To create the libraries, DNA samples (5 ng/µL) will be amplified using a primer, encoding the hypervariable regions V2-4-8 and V3-6, 7-9 of the bacterial 16 S rRNA using the 16 S Metagenomics Kit (Thermo Fisher, Waltham, MA, USA). The amplicons obtained will be purified using AMPure^®^ XP beads (Beckman Coulter, Pasadena, CA, USA) and, subsequently, the libraries will be created using the Ion Plus Fragment Library kit (Thermo Fisher, Waltham, MA, USA) and the Ion Xpress Barcode Adapters 1–16 kit (Thermo Fisher, Waltham, MA, USA) to add barcodes to purified amplicons. The libraries will be purified using AMPure^®^ XP beads (Beckman Coulter, Pasadena, CA, USA) and quantified by fluorescence. Sequencing of the libraries will be carried out using an Ion S5 platform. The results of composition, diversity, and richness will be analyzed using the QIIME2 platform, and functionality through the PICRUST software package.

#### 2.13.4. Analysis of Intestinal Permeability

Plasma levels of zonulin will be measured in duplicate using an ELISA commercial kit (IDK Zonulin ELISA, Immunodiagnostik AG, Bensheim, Germany), according to the manufacturer’s protocol.

#### 2.13.5. Determination of Endotoxemia Markers

LPS will be measured by using the Limulus Amebocyte Lysate Chromogenic Endpoint Assay (HycultBiotech, Uden, The Netherlands), following the manufacturer’s instructions.

#### 2.13.6. Determination of Inflammatory Markers

Markers of inflammation (IL-6, TNF-a, IL-1b, and IL-10) will be determined in serum by multiplex assay (Multiplex Human Cytokine MAGNETIC Panel, HCYTOMAG-60K, Merck Millipore, Burlington, MA, USA).

#### 2.13.7. Analysis of SCFAs in Fecal Samples by Gas Chromatography (GC) Coupled with a Flame-Ionization Detector

The levels of SCFAs in stool samples will be determined by GC coupled with a flame-ionization detector, as described elsewhere [22]. Briefly, 20 mg of fecal samples will be homogenized in 200 µL of distilled water and, subsequently, 100 μL of homogenized fecal samples will be mixed with 40 mg of sodium chloride, 20 mg of citric acid, 40 μL of 0.1 M hydrochloric acid, and 200 μL of butanol: tetrahydrofuran: acetonitrile (50:30:20). Samples will be vigorously vortexed and centrifuged at 14,870× *g* at room temperature for 10 min. The supernatant will be transferred to a new plastic tube, and 200 μL of a benzyl alcohol–pyridine mixture (3:2) and 100 μL DMSO will be added, and then vortexed for 5 s. Then, 100 μL of benzyl chloroformate will be added. To release the gases generated by the reaction, the tube lid will be kept open for 1 min. The tube will then be closed, and vortexed. After derivatization, 200 μL hexane will be added to the reaction mixture, and the sample vortexed for 5 min followed by a centrifugation step at 21,000× *g* for 2 min. Subsequently, 100 μL of derivative extract (upper hexane layer) will be transferred to a glass insert, and 5 μL injected in the GC-QqQ/MS in a split ratio of 25:1 using a fused-silica capillary DB-23 column Agilent 60 m × 0.25 mm (internal diameter) coated with a 0.15 μm thick layer of 80.2% 1-methylnaphatalene. Nitrogen will be used as the carrier gas at 1 mL/min (held for 4 min), reduced to 0.8 mL/min (held for 1 min) and then 0.6 mL/min (held for 1 min), and finally increased to 1 mL/min. The temperature of the FID detector will be adjusted and maintained at 260 °C, and the flow rates of H_2_, the air, and the make-up gas N_2_ to 30 mL/min, 350 mL/min, and 25 mL/min, respectively. The initial oven temperature will be set at 100 °C (held for 2 min), then increased to 200 °C at a rate of 15 °C/min, and finally maintained at 200 °C for 5 min. The identity of the SCFAs in the fecal samples will be confirmed through the comparison of their retention times and their mass spectra with those of the analytical commercial SCFA standards.

#### 2.13.8. Analysis of Plasma SCFA Levels by Ultra-High Performance Liquid Chromatography Tandem Mass Spectrometry (UHPLC-MS/MS)

For SCFA determination in plasma, 50 μL of plasma samples will be mixed with 10 μL of internal standard mixture (sodium acetate-13C2, propionic acid-d6 and butyric 1,2-13C2 acid) and 990 μL of methanol:water (50:50) mixture. Samples will be vortexed for 5 min and centrifuged for 5 min at 15,000 rpm and 4 °C. A volume of 80 μL of the supernatant will be mixed with 10 μL BHA 0.1 M and 10 μL EDC 0.25 M, vortexed and incubated at RT for 1 h in darkness to induce acid derivatization. After the incubation, serum extract will diluted 20-fold in methanol:water (50:50). Then, 200 μL of diluted sample will be extracted by 600 μL of diethyl ether through 10 min of vigorous shaking and centrifugation for 5 min at 20,000× *g* and 4 °C. After the centrifugation, 40 µL of the upper organic layer will be transferred and evaporated to dryness under a N_2_ flow and reconstituted in 200 µL of methanol:water (50:50) for UHPLC-MS/MS analysis.

The quantification of SCFA (acetic acid (AA), propionic acid (PA), butyric acid (BA), isobutyric acid (IBA), valeric acid (VA) and isovaleric acid (IVA)) will be performed by UHPLC coupled to triple quadrupole mass spectrometry using an UHPLC 1290 Infinity II Series coupled to a QqQ/MS 6490 Series (Agilent Technologies, Sta. Clara, CA, USA).

The chromatographic separation will be carried out with a gradient elution on a Kinetex polar C18 (100 × 2.1 mm, 2.6 µm) (Phenomenex, Torrance, CA, USA) column. The mobile phase will consist of 0.1% formic acid in water with 10 mM of ammonium formate (A) and 0.1% formic acid in methanol:2-propanol (9:1; *v*/*v*) (B). The gradient is as follows: 0 min 32% B, 4.6 min 60% B, 5.5 min 65% B, 7 min 98% B and 9 min 98% B, flow rate 0.3 mL/min, column temperature at 45 °C and injection volume 1 µL. The mass spectrometer operates via positive electrospray ionization, and data will be acquired in Multiple Reaction Monitoring (MRM) mode. Commercial standards will be used for the assignment and quantification of SCFA.

#### 2.13.9. Assessment of Postoperative Follow-Up and Recovery

Patients will be carefully monitored for any signs of anastomotic leak and surgical site infection symptoms (fever, leukocytosis, severe abdominal pain, etc.) within 30 days after surgery. Diagnosis of anastomotic leakage will be confirmed by radiological examination (CT scan or MRI), clinical examination, detection of serum biomarkers (CRP and procalcitonin), and post-operative endoscopy (if required). Post-operative surgical site infections will be assessed using the ASEPSIS score system. In addition, the recruited patients will be followed up for at least one year in order to evaluate recurrences of CRC, response to adjuvant therapy, and overall/disease-free survival.

Subjects will be followed up by phone every week during the intervention and for a month after surgery, and every month until the completion of the trial. Data collected from participants that withdraw from the study will be retained following the Office of Human Research Protections. No financial incentives will be provided to any of the participants.

### 2.14. Data Management

All the data generated from the project will be entered by two independent researchers of the team into an excel sheet. Files will then be double-checked for potential errors by a third independent researcher. Only individuals authorized by the Principal Investigator will have access to the data. Files will be uploaded to a clinical trial repository (e.g., Vivli, https://vivli.org/ (accessed on 20 April 2022)) and retained for up to 15 years after the end of the project.

### 2.15. Statistical Methods

The analyses of this study will follow the intention-to-treat (ITT) principles. All statistical analyses will be performed with SPSS v.22.0 software (SPSS Inc., Chicago, IL, USA), considering a level of *p* < 0.05 as statistically significant. Normality of distributions will be assessed using the Shapiro–Wilk test. A descriptive analysis of the variables will be carried out with punctual estimation and a confidence interval for 95% of security, treating the continuous variables as means, standard deviation, or medians according to the distribution of the variable. Categorical variables will be presented in frequencies and percentages. Continuous variables will be compared by Student’s “t” test and by analysis of variance (ANOVA), depending on the existence of 2 or more groups in each comparison. The association between categorical variables will be analyzed using the chi-square test. Studies of correlations between the variables will be carried out using the Pearson or Spearman test based on the normality of the analyzed variables.

Per protocol analysis will be performed within participants that fulfill the intervention. This approach will be used only for the primary outcomes and will be treated as a secondary analysis. In the case of a relevant number of missing values, multiple imputation or pattern mixture models will be used.

## 3. Discussion

To the best of our knowledge, this is the first study protocol evaluating the impact of a pre-surgical nutritional intervention based on high-fiber intake with high levels of PUFAs on altered gut microbiota and its relationship with post-surgical complications, especially anastomotic leaks and site infections. Previous studies on human subjects have demonstrated the modulation of the microbiota through dietary interventions, even in the short term [23,24,25]. In this direction, the consumption of diets rich in PUFAs have been shown to modulate the gut microbiota composition, favoring the growth of protective bacteria such as *Bifidobacteria* and *Lactobacilli*, while reducing the presence of pathobionts such as *Porphyromonas* spp. after 6 weeks of treatment [20,26,27]. Thus, our hypothesis is that the intake of a fiber-rich diet with high levels of PUFAs prior to CRC surgery could lead to a modified, more balanced gut microbiota, which could result in a lower number of complications after surgery.

## Figures and Tables

**Table 1:** Assessment schedule.

	STUDY PERIOD						
	Enrolment	Allocation	Post-Allocation				Close-Out
TIMEPOINT	t_−1_	t_0_	t_1_	t_2_	t_3_	t_4_	t_5_
ENROLLMENT:							
Eligibility screen	X						
Informed consent	X						
Inclusion criteria	X						
Exclusion criteria	X						
Allocation		X					
INTERVENTIONS:							
High-fiber diet rich in PUFAs			X	X			
Standard nutritional recommendation			X	X			
ASSESSMENTS:							
Anthropometric measurements	X						
Medication	X						
Food Frequency Questionnaire (FFQs)	X						
Tumor biopsies		X		X			X
Fecal samples			X	X			X
Blood samples			X	X			X
Anastomotic leak					X		
Surgical site infections					X		
CRC recurrence						X	
Response to adjuvant therapy						X	
Disease-free survival						X	
Quality of life questionnaire						X	

t_−1_: At baseline colonoscopy; t_0_: At baseline screening colonoscopy; t_1_: Four weeks pre-surgery intervention; t_2_: Day before surgery; t_3_: 30 days post-surgery; t_4_: 1–12 months after surgery (follow-up); t_5_: 12 months after surgery.

## Data Availability

Not applicable.

## Supplementary Materials

The following supporting information can be downloaded at: https://www.mdpi.com/article/10.3390/jcm11133613/s1, Table S1: Administrative Information; Table S2: Methods: Monitoring. Table S3: Ethics and dissemination. Annex S1: Informed consent form and other related documentation. Annex S2: SPIRIT 2013 Checklist.
