# Supplementary material for: Gut Microbiome Modification through Dietary Intervention in Patients with Colorectal Cancer: Protocol for a Prospective, Interventional, Controlled, Randomized Clinical Trial in Patients with Scheduled Surgical Intervention for CRC
Source: J Clin Med. 2022 Jun 22;11(13):3613. doi: 10.3390/jcm11133613 (PMC9267451; doi:10.3390/jcm11133613)
Supplement: Supplementary file 1 [file jcm-11-03613-s001.zip › Annex S1 Patient Information Sheet.pdf]

Annex S1. Informed consent form and other related documentation

HOJA DE INFORMACIÓN PARA PARTICIPANTES EN ESTUDIOS DE INVESTIGACIÓN CON MUESTRAS

Titulo del estudio: "MODIFICACIÓN DE MICROBIOMA INTESTINAL MEDIANTE INTERVENCIÓN DIETÉTICA EN PACIENTES CON CÁNCER COLORRECTAL (CCR) Y PREVISIÓN DE CIRUGÍA "
Investigadores Principales, servicio/unidad y centro: Bruno Ramos Molina, Instituto Murciano de Investigación Biosanitaria (IMIB); y José Gil Martínez, Hospital Clínico Universitario Virgen de la Arrixaca (HCUVA).
Nº de versión y fecha: VERSIÓN 1 (21.1.21)

INTRODUCCIÓN

Considerando la enfermedad o proceso que usted padece , nos dirigimos a usted para informarle sobre un estudio en el que se le invita a participar. Nuestra intención es que reciba la información correcta y suficiente para que pueda evaluar y juzgar si quiere o no participar en este estudio. Antes de decidir si quiere participar o no, le rogamos lea detenidamente este documento que incluye la información sobre este proyecto. Puede formular todas las preguntas que le surjan y solicitar cualquier aclaración sobre cualquier aspecto del mismo. Nosotros le aclararemos las dudas que puedan surgir en cualquier momento. Además, puede consultar con las personas que considere oportuno.

El proyecto cuenta con el informe favorable de un Comité de Ética de la Investigación acreditado en España.

PARTICIPACIÓN VOLUNTARIA

Debe saber que su participación es voluntaria y que puede decidir no participar o cambiar su decisión y retirar el consentimiento en cualquier momento, sin que por ello se altere la relación con su médico ni se produzca perjuicio alguno en su tratamiento.

PROPÓSITO Y PROCEDIMIENTOS DEL ESTUDIO:

Existe suficiente evidencia como para pensar que la microbiota intestinal (microorganismos presentes en el intestino) es decisiva a la hora de determinar el desarrollo y evolución de los tumores de colon. Somos conocedores de la importancia de la dieta a la hora de modular esta flora intestinal y determinar el pronostico de este tipo de pacientes. Se propone desarrollar un estudio sobre los efectos de la dieta en la modificación de la microbiota en pacientes con tumoración colorrectal. Se incluirán en el estudio a pacientes de ambos sexos El estudio consiste en analizar de forma prospectiva a estos pacientes tras someterse a una intervención nutricional consistente en el aporte de alimentos ricos en fibra y ácidos grasos poliinsaturados, (al comienzo del estudio cada participante recibirá una hoja informativa nutricional con los alimentos indicados

PROCEDIMIENTOS DE OBTENCIÓN DE MUESTRAS, MOLESTIAS Y POSIBLES RIESGOS:

Algunas de las muestras se obtienen durante el seguimiento habitual de su enfermedad o proceso; otras son solicitadas porque son necesarias para cumplir con los objetivos de este estudio. A continuación le explicamos cuáles son y los riesgos asociados a los procedimientos utilizados para su obtención .

Todos los pacientes con indicación de colonoscopia diagnóstica de CCR aportarán una muestra de heces recogida antes de la preparación de limpieza intestinal. En caso de CCR se realizará toma de muestra de heces

y tejido durante la exploración endoscópica y se obtendrá muestra de sangre. Todos los pacientes serán debidamente informados de la naturaleza y objetivo del estudio. En caso de aceptación a participar los pacientes se dividirán por orden de inclusión en el estudio, en dos brazos en una proporción 1:1. Un brazo control en el que los pacientes realizarán preparación preoperatoria intestinal habitual mediante lavado intestinal con administración de tratamiento empírico habitual basado en antibioterapia oral la tarde de antes a base de Neomicina y Metronidazol más Amoxi-Clavulánico intravenoso preoperatorio y otro grupo de pacientes que será sometido a intervención nutricional y en los que desde el diagnóstico endoscópico hasta la intervención quirúrgica el paciente tomará una dieta específica rica en fibra y ácidos grasos polinsaturados durante 3 semanas. A su vez, el paciente realizará la preparación preoperatoria intestinal del mismo modo que el brazo control.

El proyecto cumplirá en todo momento los principios fundamentales establecidos en la Declaración de Helsinki.

Se realizará una toma de muestra de heces y sangre el día de inicio del tratamiento y antes de iniciar la dieta. La toma de muestras se repetirá el día 15 y 30 de haber comenzado la dieta. Se obtendrán 3 muestras de sangre y la cantidad extraída en cada análisis será de 20-25 ml de sangre. Para la mayoría de las personas, las punciones con agujas para la extracción de sangre no suponen ningún problema. Sin embargo, en ocasiones, pueden provocar hemorragias, hematomas, molestias, infecciones y/o dolor en el punto de extracción de sangre. También puede sentirse mareado.

A su vez, se realizará una evaluación clínica completa de los pacientes, que incluirá el registro de datos antropométricos (altura, peso, índice de peso corporal, cintura, presión arterial), información sobre la medicación del paciente (el consumo de antibióticos es motivo de exclusión por su impacto en la microbiota intestinal), y evaluación de la ingesta nutricional utilizando cuestionarios de frecuencia alimentaria (FFQ). La composición de la microbiota intestinal se determinará en las muestras de heces y tejido tumoral mediante secuenciación y además, se determinarán marcadores de inflamación y de permeabilidad intestinal.

Las biopsias realizadas en el curso del estudio serán las correspondientes para todas las tumoraciones de colon en la práctica clínica habitual. Una biopsia es un fragmento de tejido tumoral que se extrae para estudiarlo viendo las células con un microscopio o para hacer pruebas para buscar moléculas concretas. . Puede haber ciertos riesgos, dependiendo de la localización del tumor que se está biopsiando, como la punción de un órgano cercano. Su médico del estudio comentará estos aspectos con usted.

Las muestras y los datos asociados se mantendrán bajo las condiciones de seguridad adecuadas y se garantiza que los sujetos no podrán ser identificados a través de medios considerados razonables por personas distintas a las autorizadas.

Es posible que sea necesario algún dato o muestras adicionales. En ese caso, su médico se pondrá en contacto con usted para solicitarle de nuevo su colaboración. Se le informará de los motivos y se le solicitará de nuevo su consentimiento (ver opción sí/no al final de la hoja).

Si ha decidido participar en el estudio, deberá:

- Seguir las modificaciones dietéticas suministradas por el grupo investigador y realizar un registro sobre su estado de salud, consumo de alimentos, actividad física, consumo de tabaco y alcohol tanto al comienzo como durante la duración del estudio.

- Recibir periódicamente consejos y material educativo sobre la alimentación saludable que usted deberá seguir tal como se le asigne desde el principio del estudio y recibir alimentos gratuitos para su consumo. Los consejos los recibirá de manera individual o grupal.

- Facilitar que se le realice una historia clínica, se le tome la tensión arterial, se le mida el peso, la talla y diámetros de cintura y cadera, se le realicen extracciones de sangre y suministrar muestras de heces y tejido tumoral para realizar determinaciones bioquímicas y metabólicas en las revisiones indicadas.

BENEFICIOS ESPERADOS:

No se espera un beneficio directo por su participación en el estudio. No obstante, los conocimientos obtenidos gracias a los estudios llevados a cabo a partir de sus muestras y de muchas otras pueden ayudar al avance médico y, por ello, a otras personas. No percibirá ningún beneficio económico por la donación de las muestras y la cesión de los datos proporcionados, ni tendrá derechos sobre posibles beneficios comerciales de los descubrimientos que puedan conseguirse como resultado de la investigación efectuada.

LUGAR DE ANÁLISIS Y ALMACENAMIENTO DE LAS MUESTRAS:

Durante el desarrollo del estudio sus muestras pueden ser analizadas en diversos laboratorios y se mantendrán almacenadas durante 5 años, en previsión de que fuera necesario repetir algún análisis adicional relacionado con los objetivos del estudio. Durante este proceso el responsable de las muestras será el investigador/promotor del proyecto.

INFORMACIÓN SOBRE EL DESTINO DE LAS MUESTRAS Y USO FUTURO

Una vez finalizado el estudio, las muestras sobrantes serán destruidas, a no ser que usted consienta para que puedan ser almacenadas y utilizadas en futuras investigaciones. La finalidad del almacenamiento de estas muestras es que sean utilizadas en proyectos de investigación en el futuro.

Tanto el almacenamiento durante la realización de la investigación como destino de las muestras al término de la misma será el Biobanco del IMIB (perteneciente al Registro Nacional de Biobancos del Instituto de Salud Carlos III con número de referencia PT17/0015/0038). Desde allí se cederán para proyectos autorizados, posiblemente también en el extranjero, previo dictamen favorable del comité científico y del comité de Ética del Biobanco. Usted podrá dirigirse al biobanco para obtener información de los proyectos en los que se hayan utilizado sus muestras. El paciente deberá firmar el consentimiento general de donación al Biobanco del IMIB. Los datos que se deriven de la utilización de estas muestras en futuras investigaciones se tratarán del mismo modo que el resto de datos que se obtengan durante este estudio (ver apartado de confidencialidad).

DERECHO DE REVOCACIÓN DEL CONSENTIMIENTO

Si cambiara de opinión en relación con la donación de las muestras biológicas y la cesión de los datos proporcionados, tiene derecho a solicitar su destrucción o anonimización, a través de su médico/investigador/investigador principal de la colección/ biobanco. No obstante, debe saber que los datos que se hayan obtenido en los análisis realizados hasta ese momento podrán ser utilizados para los fines solicitados y podrán conservarse en cumplimiento de las obligaciones legales correspondientes.

CONFIDENCIALIDAD/PROTECCIÓN DE DATOS PERSONALES:

Sus muestras estarán asociadas a un código (codificadas) Sólo personal autorizado (personal con perfil biosanitario asociado al proyecto) podrá relacionar la información derivada de los análisis realizados con información sobre su identidad.

A este estudio les son plenamente de aplicación la Ley Orgánica 3/2018 de 5 de diciembre, de Protección de datos de carácter Personal y garantía de los derechos digitales y el Reglamento (UE) 2016/679 del Parlamento europeo y del Consejo de 27 de abril de 2016 de Protección de Datos (RGPD). Por ello, es importante que conozca la siguiente información:

- Sus datos personales serán tratados con la finalidad indicada en el documento objeto de firma y serán conservados durante los años necesarios para cumplir con la normativa vigente aplicable.

- El Responsable del Tratamiento es el Hospital Clínico Universitario "Virgen de la Arrixaca" (Área I de Salud-Murcia/Oeste), cuyo **Delegada de Protección de Datos** (DPD) es Doña **Elena García Quiñones** con dirección en Servicio Murciano de Salud, C./ Central nº 7, Edificio Habitamia I, 30100, Espinardo-Murcia (correo electrónico: **dpd-sms@carm.es**).

- La base jurídica que legitima el tratamiento es su consentimiento

- Normativa aplicable: *Reglamento (UE) n 536/2014 del Parlamento Europeo y del Consejo, de 16 de abril de 2014 , sobre los ensayos clínicos de medicamentos de uso humano, y por el que se deroga la Directiva 2001/20/CE; Ley Orgánica 3/2018, de 5 de diciembre, de Protección de Datos de Carácter Personal y Garantía de los Derechos Digitales, Ley 14/2007, de 3 de julio, de Investigación biomédica; Real Decreto Legislativo 1/2015, de 24 de julio, por el que se aprueba el texto refundido de la Ley de garantías y uso racional de los medicamentos y productos sanitarios; Ley 44/ Ley 44/2003, de 21 de noviembre, de ordenación de las profesiones sanitarias, así como la Ley 14/1986, de 25 de abril, General de Sanidad, la Ley 41/2002, de 14 de noviembre, de autonomía del paciente, y demás legislación vigente en materia sanitaria.*

- Sus datos no serán cedidos, salvo en los casos obligados por Ley o en casos de urgencia médica. No obstante, en todo momento podrá revocar el consentimiento prestado, así como ejercer sus derechos de **acceso, rectificación, supresión, oposición, limitación del tratamiento y portabilidad,** en la medida que sean aplicables, a través de comunicación escrita al Responsable del Tratamiento de la siguiente manera **concretando su solicitud, junto con su DNI o documento equivalente**:

    - **Investigador Principal del estudio**: Bruno Ramos Molina, Instituto Murciano de Investigación Biosanitaria (IMIB); y José Gil Martínez, Hospital Clínico Universitario Virgen de la Arrixaca (HCUVA).

    - **Domicilio**: Hospital Clínico Universitario "Virgen de la Arrixaca", Ctra. Murcia-Cartagena s/n, CP 30120, El Palmar-Murcia,

- Asimismo, le informamos de la posibilidad de presentar una reclamación ante la Agencia Española de Protección de Datos (C/Jorge Juan, 6 Madrid 28001) www.agpd.es

El acceso a su información personal quedará restringido al médico del estudio/colaboradores, Autoridades Sanitarias en materia de inspección, al Comité Ético de Investigación Clínica, cuando lo precisen para comprobar los datos y procedimientos del estudio, pero siempre manteniendo la confidencialidad de los mismos.

Los datos recogidos para el estudio estarán identificados mediante un código, de manera que no se incluya información que pueda identificarle, y sólo su médico del estudio/colaboradores podrá relacionar dichos datos con usted y con su historia clínica.

A partir de dichos datos se podrán elaborar comunicaciones científicas para ser presentadas a congresos o revistas científicas siempre manteniendo en todo momento la confidencialidad de sus datos de carácter personal.

Se le informa que de conformidad a lo previsto en la Disposición adicional decimoséptima de la Ley Orgánica 3/2018, de 5 de diciembre, de Protección de Datos de Carácter Personal y garantía de los derechos digitales, así como del artículo 89 del Reglamento (UE) 2016/679, en el caso que con sus datos se lleve a cabo un tratamiento con fines de investigación en salud pública y, en particular, biomédica se procederá a:

- Realizar una evaluación de impacto que determine los riesgos derivados del tratamiento en los supuestos previstos en el artículo 35 del Reglamento (UE) 2016/679 o en los establecidos por la autoridad de control. Esta evaluación incluirá de modo específico los riesgos de reidentificación vinculados a la anonimización o seudonimización de los datos.

- Someter la investigación científica a las normas de calidad y, en su caso, a las directrices internacionales sobre buena práctica clínica.

- Adoptar, en su caso, medidas dirigidas a garantizar que los investigadores no acceden a datos de identificación de los interesados. En el supuesto de que no pueda garantizarse esta separación entre los datos y el investigador, se le garantiza un compromiso expreso de confidencialidad por parte del investigador así como de no realizar ninguna actividad de reidentificación. Se adoptarán medidas de seguridad específicas para evitar la reidentificación y el acceso de terceros no autorizados.

IMPLICACIONES DE LA INFORMACIÓN OBTENIDA AL ANALIZAR LAS MUESTRAS

En el caso de que usted lo solicite, se le podrá facilitar información acerca de los estudios de investigación en los que se hayan utilizado sus muestras, así como de los resultados generales del presente estudio.

En el caso de que en este estudio se obtengan datos que pudieran ser clínica o genéticamente relevantes para usted, e interesar a su salud o a la de su familia, podrá solicitar que le sean comunicados por su médico del ensayo si así lo indica en la casilla que aparece al final de este documento. No obstante, si el paciente hubiera indicado su negativa y cuando esta información, según criterio del médico responsable, sea necesaria para evitar un grave perjuicio para su salud o la de sus familiares biológicos, se informará a un familiar próximo o a un representante, previa consulta al Comité de Ética Asistencial del centro. La comunicación de esta información se llevará a cabo por profesionales que le podrán explicar adecuadamente su relevancia y las opciones que se pudieran plantear. En caso de información genética clínicamente relevante podrá recibir el preceptivo consejo genético.

INVESTIGACIONES FUTURAS

Autorizo la posible reutilización de datos personales con fines de investigación en materia de salud y biomédica para finalidades o áreas de investigación relacionadas con este estudio.

HOJA DE CONSENTIMIENTO INFORMADO

Titulo del proyecto: "MODIFICACIÓN DE MICROBIOMA INTESTINAL MEDIANTE INTERVENCIÓN DIETÉTICA EN PACIENTES CON CÁNCER COLORRECTAL (CCR) Y PREVISIÓN DE CIRUGÍA "

Investigadores Principales, servicio/unidad y centro: Bruno Ramos Molina, Instituto Murciano de Investigación Biosanitaria (IMIB); y José Gil Martínez, Hospital Clínico Universitario Virgen de la Arrixaca (HCUVA).

Datos del participante/paciente
Nombre

____

Investigador o persona que proporciona la información
Nombre

____

1. He leído, he sido informado y comprendo el contenido de la presente hoja de Información, lo que acredito con mi firma en prueba de mi consentimiento en todo lo que en ella se contiene.

2. Entiendo que mi participación es voluntaria y gratuita y comprendo que puedo solicitar la revocación de este consentimiento en cualquier momento, sin tener que ofrecer explicaciones y sin que esto repercuta en mis cuidados médicos presentes y/o futuros.

1. Deseo que el médico del estudio me comunique la información derivada de la investigación que pueda ser relevante y aplicable para mi salud o la de mis familiares:

   ☐ SI ☐ NO Teléfono o e-mail de contacto…………………….

2. Consiento al almacenamiento y uso de las muestras y de los datos asociados para futuras investigaciones en las condiciones explicadas en esta hoja de información.

   ☐ SI ☐ NO

3. Consiento a ser contactado en el caso de necesitar más información o muestras biológicas adicionales.

   ☐ SI ☐ NO Teléfono o e-mail de contacto…………………….

Fecha: Firma del Participante/paciente

Fecha: Firma del Investigador o persona que proporciona la información

REVOCACION DEL CONSENTIMIENTO

Yo, D/Dña………………………………………………………………… revoco el consentimiento prestado en fecha y no deseo continuar participando en el estudio “MODIFICACIÓN DE MICROBIOMA INTESTINAL MEDIANTE INTERVENCIÓN DIETÉTICA EN PACIENTES CON CÁNCER COLORRECTAL (CCR) Y PREVISIÓN DE CIRUGÍA”.

Fecha: Firma del Participante/paciente

Fecha: Firma del Investigador o persona que proporciona la información
