# Supplementary material for: Gut Microbiome Modification through Dietary Intervention in Patients with Colorectal Cancer: Protocol for a Prospective, Interventional, Controlled, Randomized Clinical Trial in Patients with Scheduled Surgical Intervention for CRC
Source: J Clin Med. 2022 Jun 22;11(13):3613. doi: 10.3390/jcm11133613 (PMC9267451; doi:10.3390/jcm11133613)
Supplement: Supplementary file 1 [file jcm-11-03613-s001.zip › Supplementary tables.pdf]

Supplementary Tables

Supplementary Table S1. Administrative information

Section/item	Item No	Description
**Administrative information**		
Title	1	Gut Microbiome Modification Through Dietary Intervention in Patients with Colorectal Cancer. Protocol for a prospective, interventional, controlled, randomized clinical trial in patients with scheduled surgical intervention for CRC.
Trial registration	2a	NCT04869956
	2b	N/A
Protocol version	3	25/04/2022; v1
Funding	4	This work was funded by the Institute of Health “Carlos III” (ISCIII), and co-funded by the Fondo Europeo de Desarrollo Regional-FEDER (grant number PI20/00505). B.R-M was supported by the “Miguel Servet Type I” program (CP19/00098, ISCIII, Spain; co-funded by the Fondo Europeo de Desarrollo Regional-FEDER). M.A.M-S was supported by a PFIS contract from the ISCIII (FI21/00003, ISCIII, Spain; co-funded by the Fondo Europeo de Desarrollo Regional-FEDER), MIQ-O is recipient of a “Miguel Servet Type II” program (CPI13/00003) from ISCIII, co-funded by the Fondo Europeo de Desarrollo Regional-FEDER, Madrid, Spain and also belongs to the regional “Nicolas Monardes” research program of the Consejería de Salud (C-0030-2018, Junta de Andalucía, Spain.
Roles and responsibilities	5a	Conception and design: Jose Gil-Martinez, Bruno Ramos-Molina.<br>Intervention design: Maria Antonia Martinez-Sanchez, Andres Balaguer-Roman, Jose Gil-Martinez, Bruno Ramos-Molina.<br>Manuscript writing, review, editing, and final approval of manuscript: all authors
	5b	The trial sponsor is the Biomedical Research Institute of Murcia (IMIB), Murcia, 30120, Spain.
	5c	The Biomedical Research Institute of Murcia (IMIB) is the Sponsor of this study. The funder and the sponsor have no role in the design of this study and will not have any role during its execution, analyses, interpretation of the data, or decision to submit results.

	5d	The study management group is responsible for the day-to-day management of the study including the Principal Investigator, operations staff, statisticians, and lead clinicians. The group will meet every three months for efficient delegation of tasks, coordination of communication flow, and management of activities.<br>Steering Committee: Bruno Ramos Molina (Chair), and José Gil Martinez (Co-Chair).

N/A: Not applicable

Supplementary Table S2. Methods: Monitoring

Section/item	Item No	Description
**Methods: Monitoring**		
Data monitoring	21a	**Data Monitoring Committee:** María Antonia Martínez Sánchez, María Ángeles Núñez-Sánchez, Andrés Balaguer-Román, Alba Oliva-Bolarín, Gabriel Pujante-Gilabert, Quiteria Hernández-Agüera, Juan Egea-Valenzuela, María José Mesa-López, Antonio José Ruiz Alcaraz, Mercedes Ferrer-Gómez, María Isabel Queipo-Ortuño, José Gil-Martínez (Co-Chair), Bruno Ramos-Molina (Chair).
	21b	No formal interim analyses are planned.
Harms	22	Patient safety and potential threats to the patients will be monitored throughout the trial. No harms are expected to arise from the nutritional intervention.
Auditing	23	No audit has been planned at this time.

Supplementary Table S3. Ethics and dissemination

Section/item	Item No	Description
**Ethics and dissemination**		
Consent or assent	26b	N/A
Confidentiality	27	All information related to the study will be securely stored at IMIB. Paper documents will be stored separately. Participants' information will be treated following the current European General Data Protection Regulation (2016/679 (GDPR)) and will not be included in the electronic database.
Declaration of interests	28	The authors declare that they have no known competing interests.
Access to data	29	Only the Principal Investigator and authorized individuals by the Principal Investigator will have access to the complete dataset.
Ancillary and post-trial care	30	Participants will be able to contact the researchers during the trial and up to 3 months after the end of participation.
Dissemination policy	31a	All results obtained from the study will be published in open-access peer-review journals. Participants will be able to obtain information about the results upon request to the Principal Investigator. In addition, results will be disseminated to stakeholders by attending to relevant conferences and seminars, and the general public by organizing talks at regional level.
	31b	Authorship eligibility will follow the criteria established by the SPIRIT guidelines.
	31c	Datasets of the microbiota analysis will be delivered into an adequate data repository after the results are published.

N/A: Not Applicable
